# Use of WHO clinical stage for assessing patient eligibility to antiretroviral therapy in a routine health service setting in Jinja, Uganda

**DOI:** 10.1186/1742-6405-5-4

**Published:** 2008-02-28

**Authors:** Shabbar Jaffar, Josephine Birungi, Heiner Grosskurth, Barbara Amuron, Geoffrey Namara, Christine Nabiryo, Alex Coutinho

**Affiliations:** 1 Department of Epidemiology and Population Health, London School of Hygiene and Tropical Medicine, Keppel Street, London WC1E 7HT, UK; 2 The AIDS Support Organisation, Old Mulago Complex, PO Box 10443, Kampala, Uganda; 3 Medical Research Council/Uganda Virus Research Institute (MRC/UVRI) Uganda Research Unit on AIDS, P.O. Box 49, Entebbe, Uganda

## Abstract

In a routine service delivery setting in Uganda, we assessed the ability of the WHO clinical stage to accurately identify HIV-infected patients in whom antiretroviral therapy should be started.

Among 4302 subjects screened for ART, the sensitivity and specificity (95% CI) of WHO stage III, IV against a CD4 count < 200 × 10^6^/l were 52% (50, 54%) and 68% (66, 70%) respectively. Plasma viral load was tested in a subset of 1453 subjects in whom ART was initiated. Among 938 subjects with plasma viral load of 100,000 copies or more, 391 (42%, 95% CI 39, 45%) were at WHO stage I or II.

In this setting, a large number of individuals could have been denied access to antiretroviral therapy if eligibility to ART was assessed on the basis of WHO clinical stage. There is an urgent need for greater CD4 count testing and evaluation of the utility of plasma viral load prior to initiation of ART to accompany the roll-out of ART.

## Introduction

In developed countries, CD4 count and plasma viral load testing are essential for monitoring HIV-infected subjects and for assessing when antiretroviral therapy (ART) should be initiated [1]. ART is being scaled-up in Africa and elsewhere. Many settings do not have the resources to test CD4 counts and plasma viral load and in these areas, a patient's HIV stage is assessed on the basis of clinical criteria alone, using the World Health Organisation (WHO) HIV clinical staging. Those at WHO stage III or IV can be eligible for ART (provided that they also satisfy psychosocial and other criteria) [2,3].

There are limited data on how well the WHO HIV clinical stage criteria identifies people correctly for ART. The WHO clinical stage was associated with survival and biological outcomes, done in the pre-ART era [4-6]. A hospital-based study conducted in Cambodia reported limited added value of using CD4 count in addition to the WHO HIV clinical staging when assessing patients for ART but the study was retrospective and comprised a sick group of patients [7]. More recently, a study conducted in Rakai, Uganda, showed that 49% of patients with CD4 count <200 were classified as WHO stage I or II while 12% who had stage III or IV also had CD4 count >200, suggesting that large numbers in need of treatment would not be considered on the basis of clinical staging [8]. The Rakai study used physicians to stage patients, working in a research setting. The accuracy of the WHO clinical stage criteria are unknown in a normal health service delivery setting, where training and clinical support of clinical staff can vary and the time pressures are great. There have been no studies published examining the importance of testing plasma viral load for assessing patients for ART eligibility in Africa.

We have screened and initiated ART in a large number of subjects in a normal health service delivery setting, and here we compare the different criteria for determining ART eligibility.

## Methods

The study was based at The AIDS Support Organisation (TASO) clinic in Jinja, SE Uganda. The setting has been described previously [9,10]. In brief, TASO is a large indigenous non-governmental organisation which provides care and counselling for HIV-infected persons in the country. The organisation has 11 large centres scattered over the country and has currently in excess of 10,000 subjects on ART. TASO treatment schedules follow Ministry of Health guidelines and clinical staff have similar qualifications and experience as those of government. TASO has a clinical centre in Jinja town located in the grounds of Jinja Hospital and is the largest provider of ART in the area.

In September, 2004, TASO began providing ART as part of HIV care. Registered clients were asked to come forward for screening. Eligibility for ART was assessed over 3 visits, usually scheduled over 4–6 weeks. At the first visit, patients were examined clinically by a physician and CD4 count was tested. Most physicians were medical officers with 1–2 years experience post-qualification. Clinical criteria for receiving ART were CD4 count below 200 × 10^6 ^cells/l or advanced WHO stage III (chronic fever of unknown origin, chronic diarrhoea of unknown origin, oral hairy leukoplakia and pyomyositis) or stage IV. From February 2005, all TASO clients starting ART were invited to join a pragmatic effectiveness trial evaluating two different strategies of ART delivery (home-based versus facility-based). The trial was integrated into routine health service delivery [9,10]. Clinical management of trial participants was done by TASO staff and was identical to that in non-trial participants. Trial subjects were asked for 5 ml of blood, just prior to starting ART, in order to test plasma RNA viral load. This testing was done for research purposes in batches and the results were not provided to patients or clinicians unless either requested [9]. The TASO ART programme reached capacity and was closed to new patients in December 2006.

Blood samples for CD4 counting were delivered to the laboratory about 100 km southwest of the clinic within 36 hours of collection. Testing was done using the BD FACS Calibur, Multitest™ method (Beckton-Dickenson, San Carlos, CA). Samples for plasma viral load testing were processed and stored at the trial site within 2 hours of collection. They were transported to the laboratory and tested using the HIV-1 RNA 3.0 Assay (bDNA) (Bayer AG).

The study was approved by the ethics committees of Uganda Virus Research Institute and the London School of Hygiene & Tropical Medicine and the US Centers for Disease Control and Prevention.

## Results

Between August 2004 and December 2006, TASO invited 4321 subjects for screening to assess their eligibility for ART. Their median age (range) was 37 (18–90) years and 74% were women. Overall 4302 were examined and tested for CD4 count (the remainder 19 subjects did not return); 2254 (52%) had CD4 count < 200 × 10^6^/l of whom 1091 (48%, 95% CI 46, 50%) were classified at WHO stage I or II. The sensitivity and specificity (95% CI) of WHO stage III or IV against a CD4 count of <200 × 10^6^/l were 52% (50, 54%) and 68% (66, 70%) respectively (Table 1). Similar sensitivity and specificity were recorded for higher cut-offs of 250 and 350 CD4 count × 10^6^/l.

**Table 1:** The sensitivity, specificity, positive (PPV) and negative predictive values (NPV) of WHO clinical stage and plasma viral load against CD4 count for the assessment of eligibility to ART.

	**CD4 count × 10^6^/l (no. of subjects)**		**Sensitivity**	**Specificity**	**PPV**	**NPV**
**All subjects screened**	**<200**	**≥ 200**	**Percent (95% CI)**			
WHO clinical stage						
III, IV	1163	648	52 (50, 54)	68 (66, 70)	64 (62, 66)	56 (54, 58)
I, II	1091	1400				

	**<250**	**≥ 250**				
III, IV	1320	491	52 (50,53)	72 (70, 74)	73 (71, 75)	50 (48, 52)
I, II	1241	1250				

	**<350**	**≥ 350**				
III, IV	1515	296	48 (46–50)	74 (72–77)	84 (82–85)	34 (32–36)
I, II	1640	851				

**Subjects enrolled in the trial**	**<200**	**≥ 200**				
WHO clinical stage						
III, IV	708	83	54 (51, 56)	37 (29, 46)	90 (87, 92)	7 (6, 10)
I, II	613	49				
advanced III, IV	561	70	42 (40, 45)	47 (38, 56)	89 (86, 91)	8 (6, 10)
I, II, early III	760	62				
IV	102	17	8 (6, 9)	87 (80, 92)	86 (78, 91)	9 (7, 10)
I, II, III	1219	115				
Plasma RNA viral load, copies per ml Ω						
≥ 100,000	878	60	66 (64, 69)	55 (46, 63)	94 (92, 95)	14 (11, 17)
<100,000	443	72				
Plasma viral load, copies per ml tested within 30 days of CD4 test						
≥ 100,000	411	19	66 (62, 70)	68 (55, 80)	96 (93, 97)	16 (12, 22)
<100,000	209	41				

Ω Median (IQR) interval between the CD4 count and plasma viral load tests was 32 (28–45) days.

Note: the subjects enrolled in the trial were a subset of all subjects screened.

Recruitment into the trial began in February 2005. Eleven subjects did not meet the eligibility criteria and a remaining 1477 were invited to join; 24 refused and 1453 were recruited. One thousand and thirty-one (71%) were women. The median age was 38 (range 18–83) years, 1050 (72%) had primary school or no education, 516 (36%) were married or living with someone, and 396 (27%) were unemployed. Subjects had taken a median 1 hour (range 0.25–6) to travel to clinic. Twenty (1%) were WHO stage 1, 642 (44%) at stage II, 672 (46%) at stage III, and 119 (8%) at stage IV. Most subjects (78%) at WHO stage I or II had presented with prurigo or upper respiratory infections. Of those at WHO stage III, 160 (24%) had either chronic fever of unknown origin, chronic diarrhoea of unknown origin, oral hairy leukoplakia, or pyomyositis, 437 (65%) had pulmonary tuberculosis in the last 5 years or oral candidiasis and the remainder 75 (11%) had of variety of conditions. Plasma viral load was tested within a median 32 (IQR 28, 45) days of the CD4 count and clinical examination. The median (IQR) CD4 counts and plasma viral loads were 109 (35, 165) × 10^6^/l and 163200 (63600, 370400) copies per ml respectively. There was a weak association between CD4 count and plasma viral load (r = -0.2 overall and -0.29 if restricted to 680 (47%) subjects who had plasma viral load tested within 30 days of CD4 testing; p < 0.0001 for both, Spearman's correlation).

Of 1321 subjects with CD4 count <200 × 10^6^/l, 613 (46%, 95% CI 44, 49%) were classified as WHO stage I, II, 760 (58%, 95% CI 55, 60%) were classified as WHO stage I, II and early stage III and 1219 (92%, 95% CI 91, 94%) were classified as WHO stage I, II or III. Furthermore, of 938 subjects with plasma viral load of 100,000 copies or more, 391 (42%, 95% CI 39, 45%) were at WHO stage I or II, 507 (54%, 95% CI 51, 57%) were classified as WHO stage I, II and early stage III and 869 (93%, 95% CI 91, 94%) were classified as WHO stage I, II or III. Sixty of 132 (45%, 95% CI 37, 54%) of subjects with CD4 ≥ 200 × 10^6^/l had plasma viral load of 100,000 copies per ml or more (32% [95% CI 20, 45%] if restricted to subjects who had plasma viral load tested within 30 days of CD4 testing).

## Discussion

This study shows that about a half of subjects with WHO stage I or II, as assessed in routine health service delivery in a typical African setting, had CD4 count <200 cells × 10^6^/l. Furthermore, over 40% of subjects at this clinical stage had high plasma viral load in excess of 100,000 copies per ml. These findings confirm those reported from Rakai, Uganda, earlier this year [8]. In the many settings in Africa, where CD4 count testing is not available, initiation of ART will have been deferred in these patients until they reached a more advanced HIV clinical stage when their CD4 count will have dropped and plasma viral load increased even further. This is of concern since patients with CD4 count <200 cells × 10^6^/l have high rates of death and infections and a greater risk of serious complications when they eventually start ART [1]. The high plasma virus load could have implications for onward transmission of HIV [11]. In developed countries treatment is usually started when a patient's CD4 count is around 250 × 10^6 ^cells/l or higher and when plasma virus load is below 100,000 copies per ml [1]. There are calls now to start treatment even earlier, at around CD4 count 350 × 10^6^/l, following recent research findings suggesting significant risk to patients with CD4 counts above 200 × 10^6^/l [12].

Our study highlights the urgent need for greater CD4 count testing prior to the initiation of ART to accompany the roll-out of antiretroviral therapy so that patients can start treatment in a timely manner. Besides the obvious survival benefit, the costs of baseline CD4 count testing would probably be far less than the health services costs incurred when ART is deferred to the time when the patient has a low CD4 count. Treating patients in Africa at around the CD4 count 200 cells × 10^6^/l, or earlier, will be a major challenge as the vast majority do not know their HIV status, and the number of new HIV infections far exceeds the number being placed on treatment [3].

Our study showed more than half of the subjects with CD4 count ≥ 200 × 10^6^/l were at WHO stage III or IV. Some of these subjects may have been incorrectly classified and may not have needed antiretroviral therapy, while others may have had AIDS defining conditions despite a high CD4 count. This requires research as unnecessary early treatment may be difficult to sustain over the long term for some patients without the necessary adherence support.

Our study also showed that 45% of subjects with CD4 count ≥ 200 cells × 10^6^/l had plasma viral load of 100,000 copies or more in whom treatment should be considered. This is probably an underestimate since we measured this only in subjects in whom ART was initiated. This group of subjects will be at increased risk of progressing to disease and transmitting the virus [10,11] and testing plasma viral load prior to initiation of ART in addition to CD4 count, may provide important additional information. However, testing plasma viral load is prohibitively expensive in many settings in Africa and the utility of plasma viral load needs further investigation in unselected cohorts of subjects screened for ART.

## Competing interests

The author(s) declare that they have no competing interests.

## Authors' contributions

SJ wrote the first draft of the paper. All authors reviewed drafts critically and contributed to ideas and writing on multiple occasions.

## References

[B1] Gazzard B, Bernard AJ, Boffito M, Churchill D, Edwards S, Fisher N, Geretti AM, Johnson M, Leen C, Peters B, Pozniak A, Ross J, Walsh J, Wilkins E, Youle M (2006). British HIV Association (BHIVA) guidelines for the treatment of HIV-infected adults with antiretroviral therapy (2006). HIV Med.

[B2] World Health Organisation (2006). Antiretroviral therapy for HIV-infections in adults and adolescents: recommendations for a public health approach.

[B3] World Health Organisation (2007). Towards Universal Access: Scaling up priority HIV/AIDS in the health sector. Progress report..

[B4] Kassa E, Rinke de Wit TF, Hailu E, Girma M, Messele T, Mariam HG, Yohannes S, Jurriaans S, Yeneneh H, Coutinho RA, Fontanet AL (1999). Evaluation of the World Health Organization staging system for HIV infection and disease in Ethiopia: association between clinical stages and laboratory markers. Aids.

[B5] Malamba SS, Morgan D, Clayton T, Mayanja B, Okongo M, Whitworth J (1999). The prognostic value of the World Health Organisation staging system for HIV infection and disease in rural Uganda. Aids.

[B6] Spacek LA, Gray RH, Wawer MJ, Sewankambo NK, Serwadda D, Wabwire-Mangen F, Kiwanuka N, Kigozi G, Nalugoda F, Quinn TC (2006). Clinical illness as a marker for initiation of HIV antiretroviral therapy in a rural setting, Rakai, Uganda. Int J STD AIDS.

[B7] Lynen L, Thai S, De Munter P, Leang B, Sokkab A, Schrooten W, Huyst V, Kestens L, Jacques G, Colebunders R, Menten J, van den Ende J (2006). The added value of a CD4 count to identify patients eligible for highly active antiretroviral therapy among HIV-positive adults in Cambodia. J Acquir Immune Defic Syndr.

[B8] Kagaayi J, Makumbi F, Nakigozi G, Wawer MJ, Gray RH, Serwadda D, Reynolds SJ (2007). WHO HIV clinical staging or CD4 cell counts for antiretroviral therapy eligibility assessment? An evaluation in rural Rakai district, Uganda. Aids.

[B9] Amuron B, Coutinho A, Grosskurth H, Nabiryo C, Birungi J, Namara G, Levin J, Smith P, Jaffar S (2007). A cluster randomised trial to compare home-based with health facility-based antiretroviral treatment in Uganda: study design and baseline findings.. The Open AIDS Journal.

[B10] Jaffar S, Amuron B, Birungi J, Namara G, Nabiryo C, Coutinho A, Grosskurth H Integrating research into routine service delivery in a antiretroviral treatment programme: lessons learnt from a cluster randomised trial comparing different strategies of HIV care in Jinja, Uganda.. Trop Med Int Health.

[B11] Kalichman L, Kobyliansky E, Livshits G (2006). Characteristics of joint degeneration in hand osteoarthritis. Joint Bone Spine.

[B12] Phillips AN, Gazzard BG, Clumeck N, Losso MH, Lundgren JD (2007). When should antiretroviral therapy for HIV be started?. Bmj.

